# Exploring Training Effect in 42 Human Subjects Using a Non-invasive Sensorimotor Rhythm Based Online BCI

**DOI:** 10.3389/fnhum.2019.00128

**Published:** 2019-04-17

**Authors:** Jianjun Meng, Bin He

**Affiliations:** Department of Biomedical Engineering, Carnegie Mellon University, Pittsburgh, PA, United States

**Keywords:** brain-computer interface, BCI, electroencephalography, EEG, training effects, behavioral performance, online control, motor imagery

## Abstract

Electroencephalography based brain-computer interfaces (BCIs) show promise of providing an alternative communication channel between the brain and an external device. It is well acknowledged that BCI control is a skill and could be improved through practice and training. In this study, we explore the change of BCI behavioral performance as well as the electrophysiological properties across three training sessions in a pool of 42 human subjects. Our results show that the group average of BCI accuracy and the information transfer rate improved significantly in the third session compared to the first session; especially the significance reached in a smaller subset of a low BCI performance group (average accuracy <70%) as well. There was a significant difference of event-related desynchronization (ERD) lateralization for BCI control between the left- and right-hand imagination task in the last two sessions, but this significant difference was not revealed in the first training sessions. No significant change of *R*^2^ value or event-related desynchronization and synchronization (ERD/ERS) for either channel C3 or channel C4, which were used for online control, was found across the training sessions. The change of ERD lateralization was also not significant across the training sessions. The present results indicate that BCI training could induce a change of behavioral performance and electrophysiological properties quickly, within just a few hours of training, distributed into three sessions. Multiple training sessions might especially be beneficial for the low BCI performers.

## Introduction

Brain-computer interface (BCI) is an emerging technique providing a unique opportunity to help patients who lose the ability to control their body through the regular neuromuscular pathway (Wolpaw and McFarland, 2004; Vallabhaneni et al., 2005; He et al., 2013, 2015). BCI has been demonstrated to establish communication with the world (Kübler et al., 2001; Wolpaw and Wolpaw, 2012; Chen et al., 2015), to assist stroke rehabilitation (Ramos-Murguialday et al., 2013; Pichiorri et al., 2015; Chaudhary et al., 2016), and to probe the function of brain circuits (Min et al., 2017). The non-invasive electroencephalography (EEG) based BCI has gained considerable attention due to its relative ease of use, low cost, high time resolution, etc., compared to BCI systems based on other modalities such as functional magnetic resonance imaging (fMRI) (Weiskopf et al., 2004; Yoo et al., 2004) and magnetoencephalography (MEG) (Mellinger et al., 2007; Ahn et al., 2013). The source signals of EEG based BCI systems consist of self-modulated spontaneous EEG and evoked potential signals such as P300 (Kaper et al., 2004; Guger et al., 2009) and steady-state evoked potential (Allison et al., 2010; Chen et al., 2015). Sensorimotor rhythms (SMR) are self-modulated spontaneous signals which are commonly used; the SMR are well documented to be modulated during subjects’ self-imagination of their body movement (Pfurtscheller and Da Silva, 1999; Wolpaw and McFarland, 2004; Pfurtscheller and Neuper, 2006; Yuan and He, 2014; He et al., 2015), i.e., motor imagery (MI). MI-based BCI through SMR modulation is an important and active research area.

Currently, the MI-based BCI shows promise for clinical applications such as in daily life assistance (Millán et al., 2010) and stroke rehabilitation (Chaudhary et al., 2016). However, MI-based BCIs are still scarcely used outside laboratories because this technique is currently limited by the substantial performance variation across subjects as a result of various reasons such as motivation (Nijber et al., 2010), the user technology relationship (Ahn and Jun, 2015; Jeunet et al., 2016) and variation within subjects due to possible hypothetical causes such as the fluctuation of attentional networks (Grosse-Wentrup and Schölkopf, 2013). Furthermore, there is a portion of the subject population, who cannot gain satisfactory performance using the current techniques (Guger et al., 2003; Blankertz et al., 2010). To solve this problem, some researchers have proposed various measures to detect these subjects who belong to the category of “BCI illiterate” (Vidaurre and Blankertz, 2010). Thus, in the beginning, they could be ruled out for a particular BCI experimental design which might not fit best for them. It would allow researchers to analyze why a specific BCI technology fails in a particular population, and at the same time it would save both the subjects’ and researchers’ time and cost for an inappropriate BCI technology (Neumann and Birbaumer, 2003; Blankertz et al., 2010; Hammer et al., 2014). In order to advance the technology and overcome the bottleneck of traditional MI-based BCI, studies were conducted to improve BCI performance including developing advanced signal processing algorithms (Blankertz et al., 2008; Ang et al., 2012; Meng et al., 2015; Edelman et al., 2016), designing new experimental paradigms such as providing physical stimuli while performing motor imagination (Yao et al., 2014, 2017), improving the visual feedback (Lotte et al., 2013; Jeunet et al., 2014; Mousavi and de Sa, 2017), incorporating users’ response to feedback signals (Ferrez and Millán, 2008; Chavarriaga et al., 2014; Mousavi et al., 2017), and leveraging their past meditation experience (Cassady et al., 2014), practicing meditation before attending motor imagination experiment (Tan et al., 2014), and immersing interactions via physical devices (LaFleur et al., 2013; Donati et al., 2016; Meng et al., 2016) or virtual reality (Royer et al., 2010; Doud et al., 2011; Guger et al., 2017; Johnson et al., 2017; Coogan and He, 2018).

As a complement to the multiple studies improving BCI performance in various ways described above, the gradual improvement of skill during multiple BCI training sessions (Kübler et al., 2004; Ono et al., 2013; Wander et al., 2013) might be an alternative natural way to increase the number of people operating MI-based BCI systems. Furthermore, some of the users who were thought to be “BCI illiterate” might not actually be “BCI illiterate.” Their failure to succeed in the control of BCI might be due to the inefficiency of the experimental design, decoding algorithm, or an insufficient amount of training (Allison and Neuper, 2010). Although MI-based BCI usually requires a long training period (Blankertz et al., 2007), training might be inevitable in some clinical applications, such as stroke rehabilitation, since end users must use the BCI system intensively for a long period to promote brain plasticity. Thus, it is important to quantify the learning effect as well as the change of electrophysiological properties during multiple BCI training sessions. Until now, there are only a few studies that analyze the change of electrophysiological characteristics during the multiple learning sessions when participants learn/gain a skill. Weber et al. (2011) explored the learning of SMR neurofeedback in two groups of healthy subjects (*N* = 13 in group one; *N* = 14 in group two). They instructed the subjects to modulate their SMR without explicitly indicating the motor imagination task. Throughout a number of training sessions up to 30, they stated that the successful prediction of good SMR modulators vs. poor SMR modulators could be made after session 10 (Weber et al., 2011). Zich et al. (2015b) reported a long-term training of 14 sessions in a single subject by a mobile EEG at home; they found a trend of increasing contralateral event-related desynchronization (ERD) of brain rhythms from 8 to 30 Hz across the training sessions. Later, Zich et al. (2015a) did a follow-up study in a group of 16 naïve BCI subjects. Each of the participants performed four sessions of BCI experiments on four consecutive days, including a first session using high-density EEG and the remaining three sessions using individualized bipolar channel pairs. They acquired wireless EEG signals using the modified Emotiv device (Zich et al., 2015a). Their results showed a significant improvement in online classification accuracy from 69.1% in session 2, to 73.3% in session 4. They also found a significant change in the difference of contra- and ipsi-lateral ERD; the difference was significantly higher in session 4 than in session 2. Due to the use of individualized sparse electrode configuration for each participant, it was not possible to quantify the electrophysiological change in global topography. Cincotti et al. (2012) studied the long-term BCI training of 12 sessions in a group of eight stroke patients and found a significant spectral power change of EEG rhythms in both alpha (8–13 Hz) and beta (14–29 Hz) bands, between task and rest conditions, when comparing the pre-training and post-training condition. Kaiser et al. (2014) studied 12 BCI naïve subjects, each of whom participated in ten BCI sessions, including one screening session, six BCI training sessions, and three fNIRs sessions. They found a significant enhancement of [oxy-Hb] in fNIRS and a stronger ERD in the upper beta-frequency (24–30 Hz) band in the EEG; however, these changes were only visible in participants with a BCI classification accuracy lower than 70%. Although the subjects in the high BCI performance group (mean accuracy >70%) did show an increasing trend of online classification accuracy (see Figure 12 in Kaiser et al., 2014), no significance was reported, perhaps due to a small sample size (*N* = 5) in this group. Due to the limited number of subjects or electrode coverage in the previous studies (Cincotti et al., 2012; Kaiser et al., 2014; Zich et al., 2015a) or the non-specified motor imagery training protocols (Weber et al., 2011), it remains unclear whether and how quickly the BCI behavioral performance would change across the training. It is still inconclusive if there would be any significant changes of electrophysiological properties locally or globally, and how the change of electrophysiological properties progress accompanied by the change of behavioral performance during the training. With the above questions in mind, we performed, in this study, a detailed analysis of the change of BCI behavioral performance in a healthy subject pool of 42 people, three training sessions each, as well as the change of BCI electrophysiological characteristics across sessions such as *R*^2^ value and ERD/ERS. We hypothesize that BCI users’ behavioral performance could improve in a few training sessions, and that their electrophysiological signatures become more distinguishable at the same time.

## Materials and Methods

Each of the 42 healthy, BCI naïve subjects (18 females; 5 left-handed and all others right-handed; average age 23.3 ± 5.5 years; range, 18–50) participated in three sessions of a BCI online cursor control experiment on separate days, which resulted in a total of 126 sessions. Each session took place on a different day, with an interval from 1 day to 1 week, except for four subjects in five cases which had longer intervals than 1 week due to schedule conflicts. None of them had an interval of less than 1 day. The sessions for each subject were scheduled as early as their next available day to minimize the session intervals. All procedures and protocols were approved by the Institutional Review Board of the University of Minnesota and Carnegie Mellon University. Written informed consent was obtained from all subjects before their participation in the experiment.

### Experimental Setup

**FIGURE 1:**
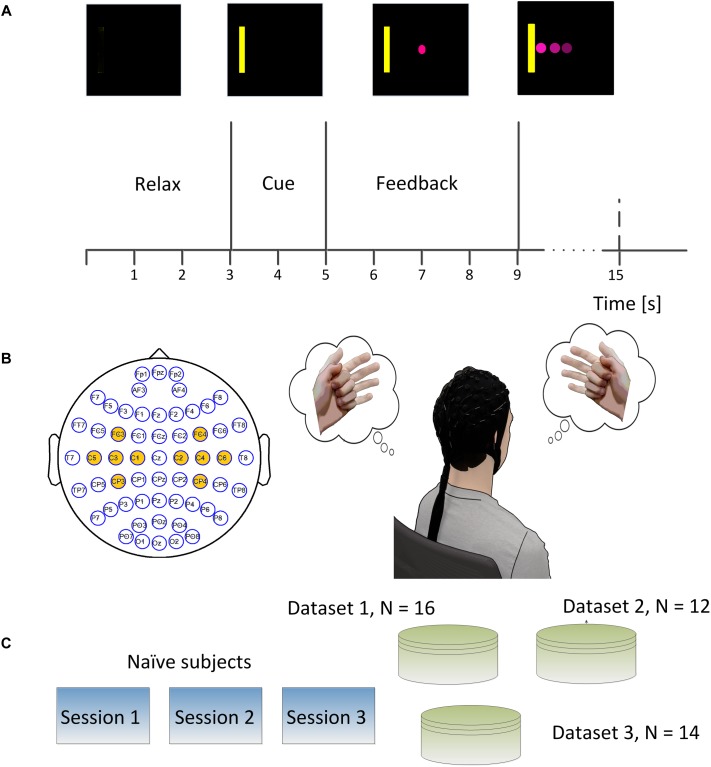
**(A)** Brain-computer interface (BCI) single trial structure. Each trial started from a blank screen of 3 s during which subjects should relax and have a short break. Then a yellow rectangle appeared either on the left side or right side of the screen at the end of second 3 and lasted for 2 s. A round pink cursor appeared at the center of screen at the end of second 5 and provided the online real time feedback. The cursor was supposed to move toward the left side or right side of the screen if subjects performed left hand or right hand motor imagination. The subjects were allowed hitting the correct target or the incorrect target within a period of 6 s, and then the cursor froze for 1 s during the post-feedback period. **(B)** The electrodes which were marked in yellow denoted the ones used for online control. The subject was supposed to imagine the left hand movement when the yellow bar appeared on the left side of the screen and imagine the right hand movement when the yellow bar appeared on the right side of the screen. **(C)** Experimental scheme of the study. Each of 42 naïve subjects participated in three sessions of online BCI control.

Electroencephalography signals were recorded in three experiments (See Figure 1C) including the first dataset in a published study (Meng et al., 2018) and the other two in another published study (Meng et al., 2017). In the first two experiments, EEG signals were recorded at a sampling rate of 1000 Hz using a 62 channels of Neuroscan SynAmps RT system (Neuroscan Inc., Charlotte, NC, United States). A bandpass filter between 0.5 and 200 Hz and a notch filter of 60 Hz was applied to the raw EEG signals. The reference was selected at the vertex, and the ground was chosen on the forehead. The impedances for all the electrodes were maintained below 5 kΩ. In the third experiment, the EEG signal was acquired at a sampling frequency of 1024 Hz using a 64 channel Biosemi Active Two EEG system and a cap with active electrodes. Similarly, a bandpass filter between 0.16 and 100 Hz and a notch filter of 60 Hz was applied to the raw EEG signal. Two electrodes near channel POZ, named CMS and DRL, were used as the reference and ground. Conductive gel (SignaGel, Cortech Solutions) was used to reduce the electrode offsets, a measure indicating signal quality, to below 20 mV for each electrode as recommended by the manufacturer.

### Experimental Design and Protocol

In the first experiment, 16 subjects participated in the study and their EEG signals were acquired using a Neuroscan system following the above-described procedures. Each subject participated in three sessions of online BCI control – left and right (L/R) cursor movement – on separate days. There were ten runs of BCI control in each of the three sessions, and each run took about 5–6 min with an interval of 1–2 min according to the subject’s willingness. There were 25 trials in each run and 250 trials in each session. The left or right targets were balanced in a block randomized way. In the second experiment, 12 subjects participated in the study following the above-described procedures, and their EEG signals were acquired using a Neuroscan system. Similarly, subjects took three sessions of BCI L/R cursor control on different days. There were four runs of BCI control in each session, and each run took about 6–8 min with an interval of 1–2 min between two runs at the subject’s willingness. There were 30 trials in each run and 120 trials in total for each session. In the third experiment, 14 subjects participated in the study following the above-described procedures and EEG signals were acquired using a Biosemi Active Two system. There were eight runs of BCI online L/R cursor control in each of the three sessions, and each run consisted of 20 BCI trials resulting in 160 trials in total for each session.

The trial structures for all the three experiments were the same (See Figure 1A). Each trial started with a blank screen, and the blank screen lasted for 3 s, which was referred to as the inter-trial interval. A yellow square served as a target cue and appeared on either the left or right side of the screen after 3 s. This target cue was displayed for 2 s to help the subject prepare for the following cursor feedback; subjects were instructed to perform motor imagination of their left- or right hand to move the cursor toward the left or right correspondingly (See Figure 1B). A round pink cursor appeared in the center of the screen after second 5. The cursor was given a mean velocity which allowed users to hit the target within 3 s if the control signal was stationary and correctly decoded at each update window (40 ms) during the whole trial; the cursor stopped if the subjects could neither hit the target nor miss the target in the duration of 6 s, i.e., an abort trial. After the feedback period, the cursor was frozen for 1 s, and this period was named the post-feedback period. Then the next trial repeated until the end of the run. All the subjects were instructed to perform the kinesthetic motor imagination from a first person perspective (Neuper et al., 2005).

### Online Signal Processing

The modulation of the power difference between channel C3 and C4 was used to control the cursor movement. The raw EEG signals of channel C3 and C4 were first spatially filtered by subtracting the average signals of its surrounding electrodes, i.e., the small Laplacian spatial filter (See Figure 1B), to remove the common noise and to obtain relatively focal activity (Hjorth, 1975; McFarland et al., 1997). The power spectra of the two channels were estimated using an autoregressive (AR) method (McFarland and Wolpaw, 2008). An autoregressive (AR) model, as shown in Eq. (1), was used to estimate the amplitudes of sensorimotor rhythm:

(1) $$y_{t}=\overset{i=p}{\underset{i=1}{\Sigma }}w_{t-i}y_{t-i}+\epsilon$$

where y_t_ is the estimated signal at time *t*, *w*_i_ is the weight coefficient and 𝜖 is the error of estimation. An 16th order AR model with a window length of 400 ms was used to calculate the online amplitude of mu rhythmic activity. The weight coefficients of the AR model were estimated by the least-squares criteria. The power spectra were updated every 40 ms. The power difference of the mu rhythm (10–14 Hz) between the two channels was stored in a buffer of the previous 30 s data and was output to a normalizer, as shown in Eq. (2):

(2) Output=(Input−Offset)*Gain⁢

where the *Input* contains the data (the above-mentioned power difference of mu rhythm) in a buffer of the past 30 s data. The normalizer applied a linear transformation to its *input* signal and transformed the *output* of power difference into a zero mean and unit variance value according to Eq. (2). Thus, the *offset* and the *gain* in Eq. (2) are the mean and multiplicative inverse of the standard deviation of the input data, respectively. The Output signal was then projected into the velocity of cursor movement. The application of BCI cursor control was realized by BCI2000 software (Schalk et al., 2004). Note that, a certain period was used to train the normalizer since *Input* data must be accumulated into the buffer. Thus, the cursor did not move for the first trial of each BCI session. The subject was still instructed to start their motor imagination when the cursor was shown on the screen although there was no feedback of cursor movement during the first trial. The first trial of each session was not considered in the analysis since it was always an abort trial.

### Evaluation of Behavioral Performance

The behavioral performance of online BCI was evaluated in terms of Percent Valid Correct (PVC) (Doud et al., 2011; Meng et al., 2016), which means that the number of hits during each run was divided by the total number of hits and misses. The number of abort trials was not considered for this metric. Since the number of abort trials was not considered in PVC, we wanted to see the trend of the number of abort trials across the training sessions. Thus, the percentage of abort trials during each run was calculated and averaged over the runs for each session. A group average over the percentage of abort trials across sessions was used to evaluate the trend for the percentage of abort trials. The skills of BCI performance could not only be evaluated by accuracy but also by efficiency such as information transfer rate (ITR) (Wolpaw et al., 2000). The ITR was calculated by accounting for the abort trials, break time and the target presentation time. The group average of ITR across sessions was used to evaluate the efficiency of BCI control. Since the cursor movement was controlled in a velocity-based mode, the average duration of hit trials is another index pertinent to the efficiency of the control. The mean duration of hit trials in each run was averaged over the runs for each session; a grand average of duration for hit trials over subjects across sessions was investigated additionally, as an extra measurement of BCI cursor control.

Riemannian class distance proposed by Lotte and Jeunet (2018) was also used to assess the performance of BCI control. The distinctiveness of the EEG patterns (represented by class Dis) in the frequency band of 10–14 Hz, from two motor imagery classes were calculated for each individual and each session. Their group average results were used to evaluate the distinctiveness of the EEG covariance matrix during BCI control. All the BCI trials during the feedback period and channels were used for the calculation of Riemannian class distance in this study.

### Calculation of *R*^2^ Value and ERD/ERS

Besides the evaluation of behavioral performance, the examination of neural electrophysiology across the learning sessions is also interesting and important. R-square (*r*^2^) value could be used to quantify how strongly the means of the two distributions (the band power of the left and right-hand imagination in this case) differ relative to the band power variance. The *R*^2^ value could be calculated at each electrode according to its definition and gave rise to a *R*^2^ topography to show how strongly the electrodes correlate with the task. In the offline analysis, the *R*^2^ values were calculated based on all the trials and only the trials in which correct targets were hit, respectively, in the frequency band of mu rhythm which was used for the online modulation. When the calculation was based on all the trials, the trials of miss and abort were included in the calculation. The *R*^2^ values were calculated for each subject and each session. Grand average *R*^2^ values and their topographies across sessions were used to evaluate the change of *R*^2^ values across sessions.

Event-related de-synchronization/synchronization (ERD/ ERS) is a frequently used metric to characterize the dynamic change of band power activities relative to the baseline period in a certain location/electrode and in a specific frequency band (Graimann et al., 2002; Pfurtscheller et al., 2006; Wolpaw and Wolpaw, 2012). There are several methods to calculate the ERD/ERS time courses. In this paper, we used a bootstrap-based method (Graimann et al., 2002) to show a time-frequency map with significant changes of ERD or ERS for specific electrodes. In general, the calculation of ERD/ERS is performed by spatially filtering the EEG signals via a small Laplacian, bandpass filtering the EEG signals, segmenting individual trials, detrending the trials, squaring the samples, and subsequently averaging over trials and sample points. The procedures can be expressed using the following steps:

(3) $$y_{ij}={\left(x_{ij}-\overset{¯}{x_{j}}\right)}^{2}$$

(4) $$A_{j}=\frac{1}{N-1}\overset{N}{\underset{i=1}{\Sigma }}y_{ij}$$

(5) $$R=\frac{1}{K}\overset{r_{0}+K}{\underset{r_{0}}{\Sigma }}A_{j}$$

(6) $${ERD}_{j}=\frac{A_{j}-R}{R}\times 100\%$$

where N is the total number of trials, x_ij_ is the jth sample of the ith trial of the bandpass filtered EEG signals and $\overset{¯}{x_{j}}$ is the mean of the _j_th sample averaged over all trials. R is the average power in the reference period [r_0_,r_0_ + K], r_0_ is the starting time point of the reference period (r_0_ is 1.5 s in Figure 1A) and K is the number of samples in the baseline reference period (*K* = 1.5 × Fs, Fs is the sampling rate, 1000 or 1024 Hz). Their functional role has been hypothesized in this way: ERD in alpha and beta band represents an event relevant and information facilitating brain state, and ERS represents an event relevant but inactive or inhibited brain state (Pfurtscheller and Neuper, 2006). Thus, they are metrics correlated to the activeness of the cortical network to a certain degree. For each subject, the last 1.5 s of the inter-trial interval was selected as the baseline period. Channel C3 and C4 were used to calculate the ERD/ERS since these two channels were used for the online control. The time course of ERD/ERS was calculated from the beginning of the inter-trial interval to 2.5 s after the feedback was shown using all the trials and only hit trials, respectively, in a session. A grand average for subjects was obtained across sessions for each control task, i.e., right-hand task, and left-hand task. Besides the commonly used ERD/ERS, ERD lateralization (Zich et al., 2017; Shu et al., 2018) calculating the difference between contra- and ipsi-lateral ERD was shown to be a useful metric to identify the neurophysiological change in both the healthy population and patients. Thus, this metric was investigated as well.

### Statistical Analysis

Statistical analysis was performed in R (R Development Core Team, 2006). Unless otherwise stated, the following statistical tests were used to report results. For the statistical analyses of behavioral performance, a linear mixed effect model (lme) was employed to evaluate the statistical significance of group performance across sessions with the *post hoc* Tukey’s tests used to correct for multiple comparisons (between sessions). These instances are noted throughout the results section. For the statistical analyses on the change of the *R*^2^ values (comprising the factors sessions: session 1 (S1), session 2 (S2), session 3 (S3), and channels: C3 and C4), ERD/ERS values on channel C3 and C4 across the three training sessions (comprising factors sessions: S1, S2, S3 and channels: C3 and C4) and ERD lateralization values across the three training session (comprising factors sessions: S1, S2, S3 and imagination task: right hand and left hand), the mixed repeated measures ANOVA was used to determine whether the change of values on the two channels or two imagination task were significant across the three training sessions. A linear mixed effect model (lme) was employed to evaluate the statistical significance of the *post hoc* test. When appropriate, a Tukey’s HSD *post hoc* test was used to correct for multiple comparisons. For all ANOVAs, Mauchly’s test was used to check the sphericity, and Greenhouse-Geisser epsilon values were used to account for the violations of sphericity.

## Results

### BCI Behavioral Performance Across Sessions

**FIGURE 2:**
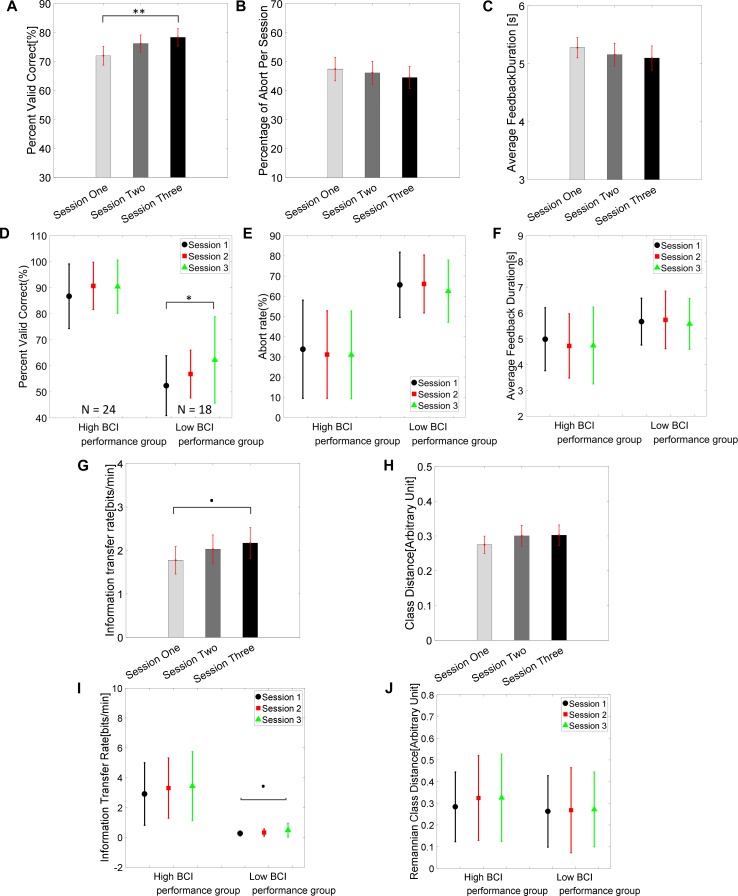
Brain-computer interface behavioral performance. **(A)** Group average PVC ± standard error of the mean (SEM) of 42 subjects across the three training sessions. The statistical analysis showed there was a significant difference of PVC between sessions 1 and 3 (^∗∗^*p* = 0.004, Tukey’s HSD *post hoc* test). **(B)** Group average percent of abort rate ± SEM across the three training sessions. **(C)** Group average feedback duration ± SEM across the three training sessions. **(D–F)** Group average PVC, abort rate, and feedback duration in high BCI performance group (average PVC across three session > = 70%, *N* = 24) and low BCI performance group (<70%, *N* = 18). **(G)** Group average information transfer rate (ITR) ± SEM across three sessions. **(H)** Group average class Riemannian distance ± SEM across three sessions. **(I,J)** Group average ITR and class Riemannian distance in high and low BCI performance groups. ^∗^*p* < 0.05.

The group average BCI performance of 42 subjects across the three training sessions are shown in Figure 2A. The average PVC of 42 subjects on the first session was 72.0 ± 3.2% and ended with 78.3 ± 3.0% on the third session. The linear mixed effect models (lme) were performed between each session pair, and Tukey’s HSD *post hoc* test was used to correct for multiple comparisons. The statistical analysis revealed that there was a significant difference between the average PVC of session 1 and session 3 (PVC: S3–S1 = 6.35%, SEM = 1.99, *Z*_value_ = 3.19, *p* = 0.004); there was no significant difference among other pairs (PVC: S2–S1 = 4.19%, SEM = 1.99, *Z*_value_ = 2.10, *p* = 0.089; PVC: S3–S2 = 2.17%, SEM = 1.99, *Z*_value_ = 1.09, *p* = 0.52). The result for the second measure of the group average abort rate is displayed in Figure 2B. The average group abort rate on the first session was 47.4 ± 4.1% and ended with 44.5 ± 3.8% on the third session. This analysis also showed that there was no significant difference among any session pairs (Abort rate: S2–S1 = -1.31%, SEM = 1.54, *Z*_value_ = -0.85, *p* = 0.67; Abort rate: S3–S1 = -2.93%, SEM = 1.54, *Z*_value_ = -1.90, *p* = 0.14; Abort rate: S3–S2 = -1.62%, SEM = 1.54, *Z*_value_ = -1.05, *p* = 0.54). The result for the third measure of group average feedback duration is illustrated in Figure 2C. The group average feedback duration was 5.28 ± 0.18 s on the first session and the result was 5.10 ± 0.21 s on the third session. Similarly, no significant difference among any session pairs was revealed by the statistical analysis (Feedback duration: S2–S1 = -0.12 s, SEM = 0.096, *Z*_value_ = -1.25, *p* = 0.422; Feedback duration: S3–S1 = -0.17 s, SEM = 0.096, *Z*_value_ = -1.85, *p* = 0.15; Feedback duration: S3–S2 = -0.05 s, SEM = 0.096, *Z*_value_ = -0.60, *p* = 0.82). We further divided each individual’s results according to their average performance in the three sessions. If their average performances were higher than 70%, they were labeled as high BCI performers. Otherwise, the remaining subjects were categorized into the low BCI performance group. Their group average results of PVC, abort rates and average feedback duration are shown separately in Figure 2D–F, respectively. There was a statistically significant difference of PVC between session 1 and session 3 in the low BCI performance group (PVC_low BCI performance_: S3–S1 = 9.89%, SEM = 3.61, *Z*_value_ = 2.74, *p* = 0.02), but not in the high BCI performance group (PVC_High BCI performance_: S3–S1 = 3.70%, SEM = 2.10, *Z*_value_ = 1.76, *p* = 0.18) even though an increasing trend can be observed in Figure 2D. The group average of ITR results are displayed in Figure 2G and the Riemannian class distances are shown in Figure 2H. Their subgroup results for the high and low BCI performers are shown in Figure 2I,J, respectively. A marginally significant improvement of ITR (ITR: S2–S1 = 0.25, SEM = 0.18, *Z*_value_ = 1.4, *p* = 0.34; ITR: S3–S1 = 0.40, SEM = 0.18, *Z*_value_ = 2.19, *p* = 0.07; ITR: S3–S2 = 0.14, SEM = 0.18, *Z*_value_ = 0.79, *p* = 0.71) was shown from session 1 to session 3 in the group average results of all 42 subjects; a close to significant level of ITR improvement was only found in the low BCI performance group (ITR_lowBCIperformance_: S3–S1 = 0.22, SEM = 0.10, *Z*_value_ = 2.23, *p* = 0.066), which is marked as a dot in Figure 2I. No significant difference of Riemannian class distance was found between any session pairs (class Dis: S2–S1 = 0.02, SEM = 0.016, *Z*_value_ = 1.59, *p* = 0.25; class Dis: S3–S1 = 0.27, SEM = 0.016, *Z*_value_ = 1.71, *p* = 0.2; class Dis: S3–S2 = 0.002, SEM = 0.016, *Z*_value_ = 0.13, *p* = 0.99).

### BCI *R*^2^ Value Across Sessions

**FIGURE 3:**
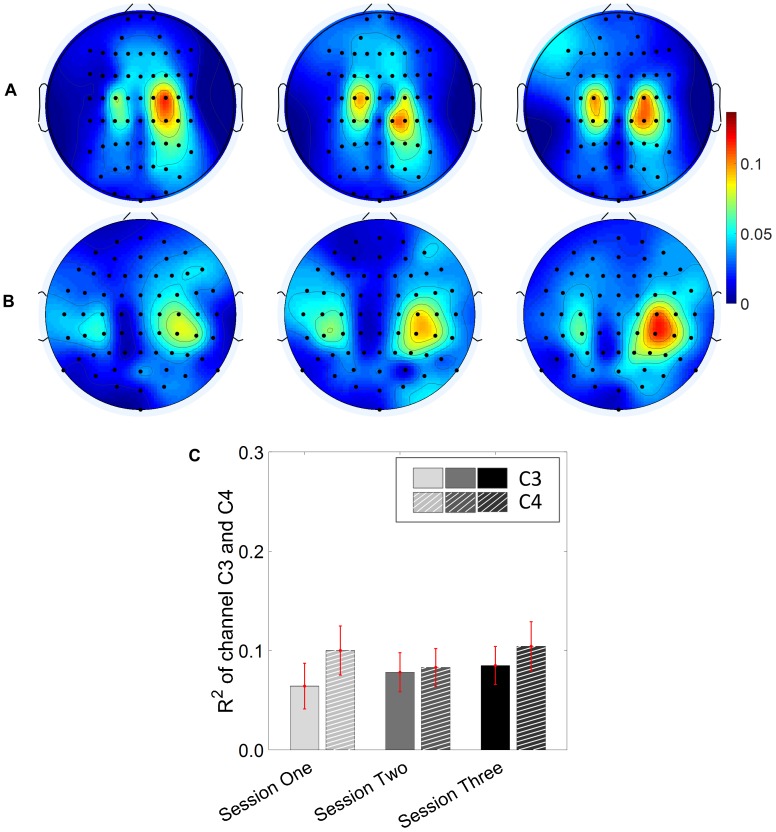
*R*^2^ value topography maps and its statistics. All of the trials are used for the calculation of *R*^2^ values. **(A)** Topography of group average *R*^2^ value across the three training sessions for subjects using Neuroscan SynAmps RT acquisition system (*N* = 28). **(B)** Topography of group average *R*^2^ value across the three training sessions for subjects using Biosemi Active Two acquisition system (*N* = 14). **(C)** *R*^2^ value of channel C3 and C4 across the three training sessions. The statistical analysis revealed no significant difference of *R*^2^ value between channel C3 and C4 on the three training sessions.

**FIGURE 4:**
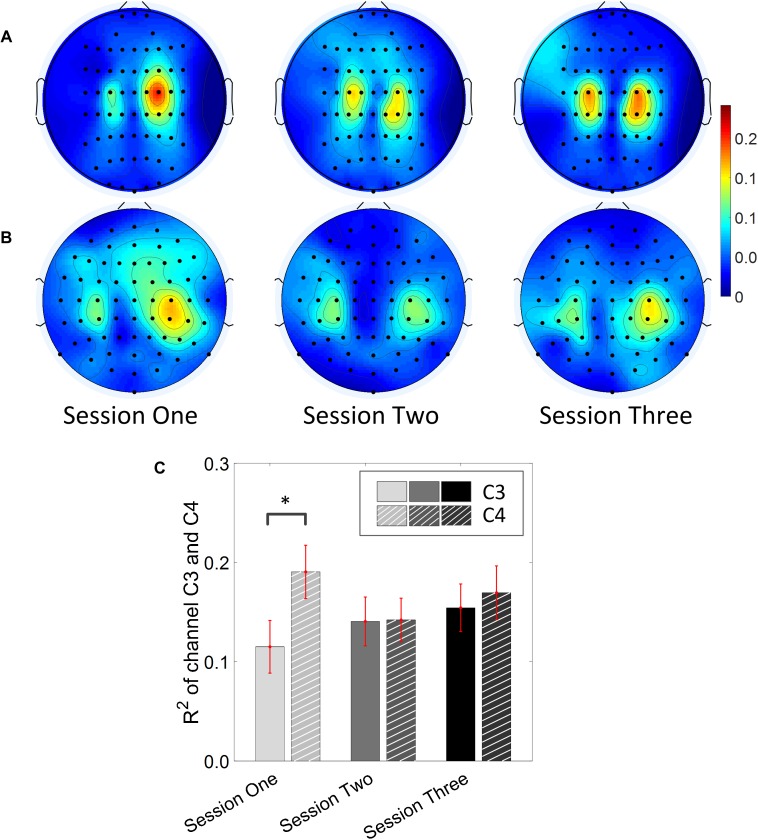
*R*^2^ value topography maps and its statistics. Only the hit trials are used for the calculation of *R*^2^ values. **(A)** Topography of group average *R*^2^ value across the three training sessions for subjects using Neuroscan SynAmps RT acquisition system (*N* = 28). **(B)** Topography of group average *R*^2^ value across the three training sessions for subjects using Biosemi Active Two acquisition system (*N* = 14). **(C)** *R*^2^ value of channel C3 and C4 across the three training sessions. The statistical analysis revealed that there is significant difference of *R*^2^ value between channel C3 and C4 on the first training session (*p* = 0.04). ^∗^*p* < 0.05.

**FIGURE 5:**
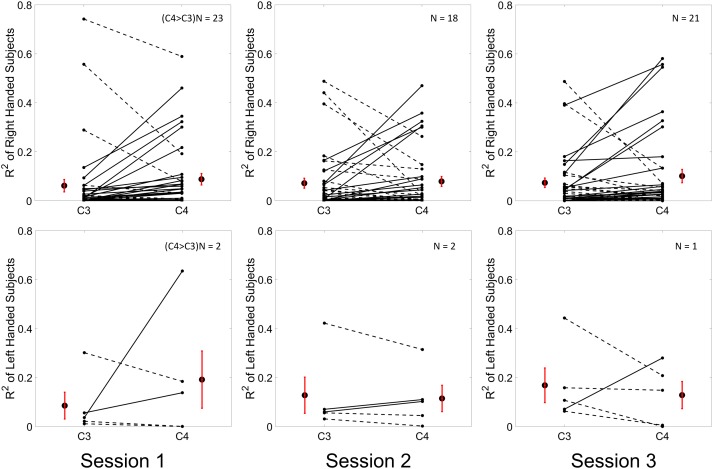
Individual and group average *R*^2^ values of channel C3 and C4 which are calculated from all of the trials across the three training sessions are shown in the first row for *N* = 37 right handed subjects and in the second row for *N* = 5 left handed subjects. The *R*^2^ values for each individual and each channel were plotted as a dot in each subplot. A solid line between the value of channel C3 and channel C4 of each individual was depicted if the value of channel C3 was smaller than the value of channel C4, otherwise, a dashed line was connected. The group average *R*^2^ values ± SEM for channel C3 and C4 were shown alongside of the individual *R*^2^ values. The results for three sessions were plotted in three columns, respectively.

Results for the group average of R-square (*r*^2^) value across the three sessions based on all the trials and only the hit trials are displayed in Figure 3, 4, respectively. The topographies of the *R*^2^ value, based on the signals acquired from the Neuroscan system (*N* = 28), are shown on the first row, Figure 3A, 4A. The topographies of the *R*^2^ value, based on the signals acquired from the Biosemi system (*N* = 14), are shown in the second row, Figure 3B, 4B. Because there are some minor differences on the electrode’s layout between the Neuroscan caps and the Biosemi caps, the topographies were plotted separately. The topographies calculated from all trials and only the hit trials, in general, displayed similar results. Yet, the *R*^2^ values calculated from only the hit trials were stronger than those calculated from all the trials. Both topographies in Figure 3, 4 indicate that channel C4 and channel C3 showed larger *R*^2^ values compared to other electrodes, and so did channel CP4 and CP3. This was expected since we used C4 and C3 as control signals, and it also fits well with the neurophysiological prior where the somatosensory cortex was beneath those electrodes. Figure 3C, 4C display the *R*^2^ value of channel C3 and C4 across the three sessions, respectively. They were distinguished by whether there was a hatch filled pattern inside the bar. A 2 (Channel: C3/C4) × 3 (sessions) ANOVA was performed to evaluate the change of *R*^2^ values with respect to factors of training and channels. This ANOVA test was conducted for the *R*^2^ values of all trials and only hit trials, respectively. While the *R*^2^ value of channel C3 seemed to show an increasing trend for visual inspection, the analysis did not show any significant difference among any session pairs (main effect channel: *F*_1,82_ = 0.49, *p* = 0.49, η^2^ = 0.005; main effect sessions: *F*_2,164_ = 1.17, *p* = 0.31, η^2^ = 0.002; channel × session interaction: *F*_2,164_ = 1.19, *p* = 0.31, η^2^ = 0.002) when analyzing the *R*^2^ values from all of the trials. However, when only hit trials were used to calculate the *R*^2^ values, a significant channel × session interaction was observed (main effect channel: *F*_1,82_ = 0.97, *p* = 0.33, η^2^ = 0.009; main effect sessions: *F*_2,164_ = 0.95, *p* = 0.39, η^2^ = 0.003; channel × session interaction: *F*_2,164_ = 3.46, *p* = 0.034, η^2^ = 0.009). *Post hoc* linear mixed effect models (lme) were performed between channel C3 and C4 in each session (S1: C4–C3 = 0.08, SEM = 0.037, *Z*_value_ = 2.02, *p* = 0.04; S2: C4–C3 = 0.001, SEM = 0.033, *Z*_value_ = 0.046, *p* = 0.96; S3: C4–C3 = 0.015, SEM = 0.036, *Z*_value_ = 0.42, *p* = 0.67), the statistics showed that there was a significant difference of the *R*^2^ value between channel C3 and C4 on the first training session in Figure 4C if only hit trials were considered, but this difference disappeared after the first training session. There was no statistical significance in Figure 3C when all the trials were included for the calculation and statistics. The individual and group average *R*^2^ values of channel C3 and C4 were divided into subgroups according to subjects’ handedness and are shown in Figure 5. The results (calculated from all the trials) for the *N* = 37 right-handed subjects and *N* = 5 left-handed subjects across three training sessions are displayed in the first and second row of Figure 5, respectively. There was no statistically significant difference between the group average *R*^2^ values of channel C3 and C4 in neither the right-handed nor the left-handed subject group. In the right-handed group, the number of subjects with a higher *R*^2^ value in channel C4 were 23/37 in Session 1, 18/37 in Session 2, 21/37 in Session 3. Yet in the left-handed group, the number of subjects with a higher *R*^2^ value in channel C4 were 2/5 in Session 1, and 1/5 in Session 2, 1/5 in Session 3. Note that there were only five left handed subjects in this study, and thus there might be little chance for the limited number of subjects to pass the significance test.

### Change of ERD/ERS Across Sessions

**FIGURE 6:**
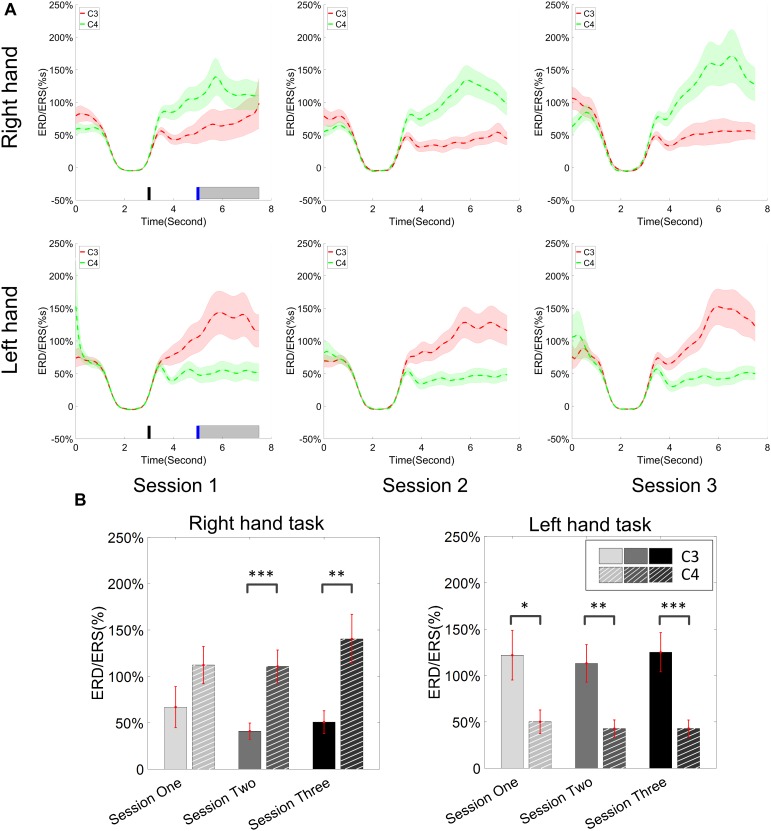
ERD/ERS value and its statistics. All of the trials were used for the calculation of ERD/ERS values. **(A)** The time varying group average of ERD/ERS value for the right hand task (the upper row) and left hand task (the bottom row) across the three training sessions. The target cue appeared at the end of second 3 and the cursor feedback began at the end of second 5. **(B)** The grand average ERD/ERS values during the period of [5–7.5]s, which was marked in gray bar area in subplot **(A)**, were compared for right hand tasks and left hand tasks, separately. The statistical analysis showed a significant difference between channel C3 and C4 during each session for the left hand task, on sessions 2 and 3 for the right hand task; no significance was found among the session pairs across the training. ^∗^*p* < 0.05, ^∗∗^*p* < 0.01, ^∗∗∗^*p* < 0.001.

**FIGURE 7:**
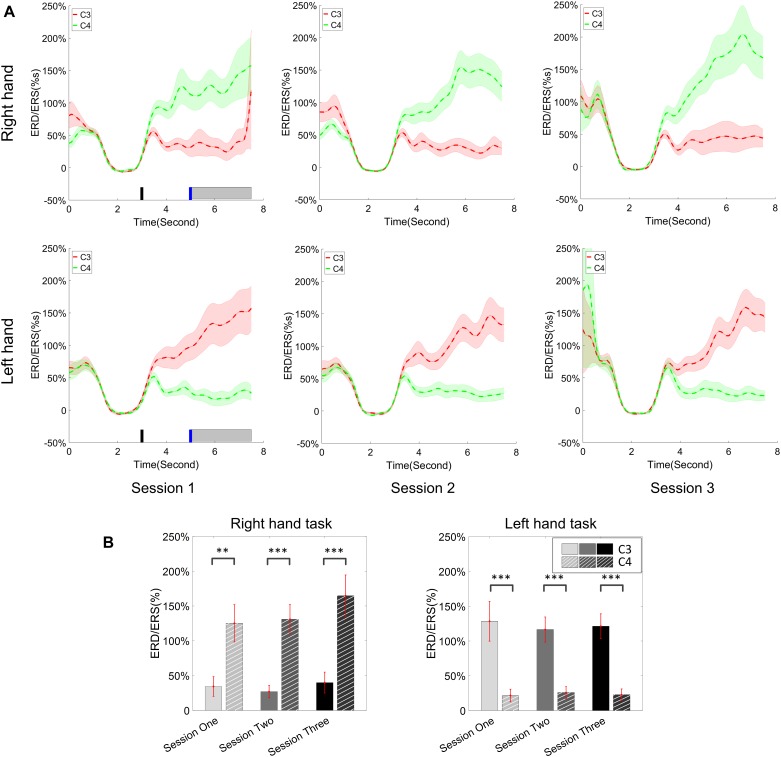
ERD/ERS value and its statistics. Only the hit trials are used for the calculation of ERD/ERS values. **(A)** The time varying group average of ERD/ERS value for the right hand task (the upper row) and left hand task (the bottom row) across the three training sessions. The target cue appeared at the end of second 3 and the cursor feedback began at the end of second 5. **(B)** The grand average ERD/ERS values during the period of [5–7.5]s, which was marked in gray bar area in subplot **(A)**, were compared for right hand tasks and left hand tasks, separately. The statistical analysis showed a significant difference between channel C3 and C4 during each session; no significance was found among the session pairs across the training. ^∗∗^*p* < 0.01, ^∗∗∗^*p* < 0.001.

**FIGURE 8:**
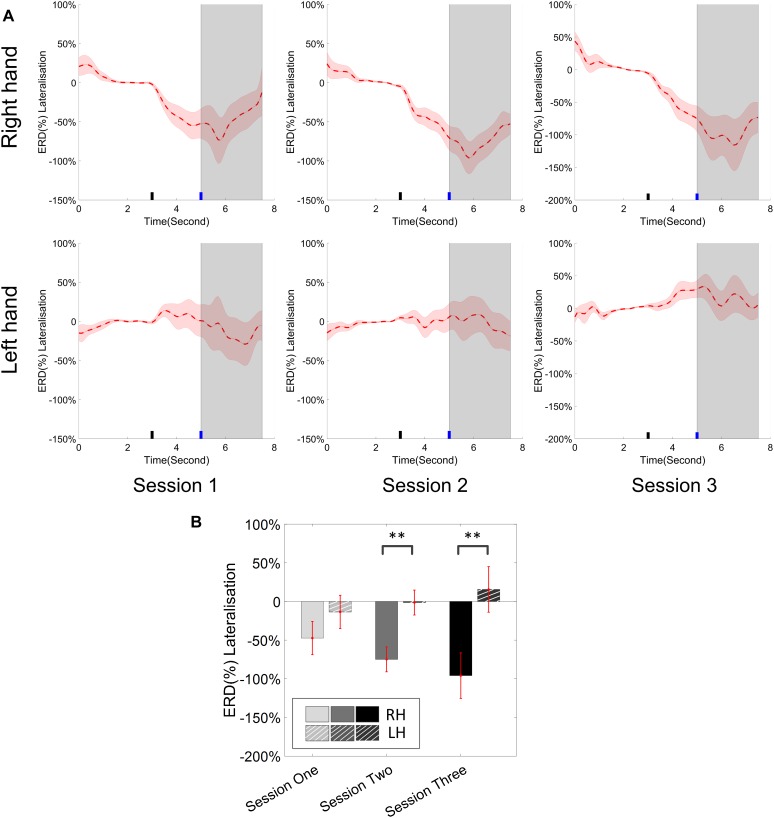
**(A)** Group average ERD% lateralization (difference between contra- and ipsilateral ERD%, calculated from all the trials) with respect to the hand task, across the three training sessions. The shaded red areas represented the standard error of the mean. The target cue appeared at the end of second 3 and the cursor feedback began at the end of second 5. **(B)** The grand average ERD% lateralization values during the period of [5–7.5]s, which was marked in gray area in subplot **(A)**. Error bars represented the standard error of the mean. ^∗∗^*p* < 0.001.

**FIGURE 9:**
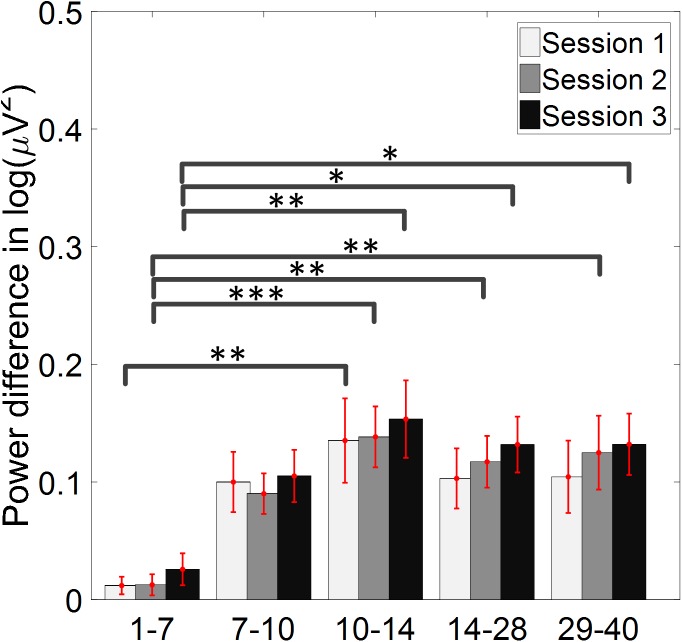
Log power spectra difference between channel C3 and channel C4 of five frequency bands across three training sessions. The log power difference between ipsilateral and contralateral hemisphere, with respect to the direction of movement imagination, was shown. There was no significant difference across the three training sessions in any of the five frequency bands. ^∗^*p* < 0.05, ^∗∗^*p* < 0.01, ^∗∗∗^*p* < 0.001.

**FIGURE 10:**
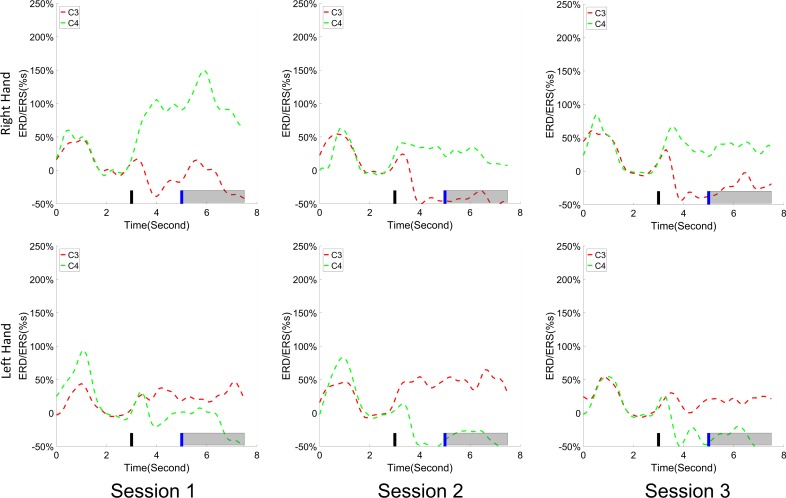
An individual example (Subject 3) of the time varying average ERD/ERS value for the right hand task (the first row) and the left hand task (the second row) across the training sessions. Shown is the similar convention to the group average results. A strong ERD on the contralateral hemisphere and a moderate ERS on the ipsilateral hemisphere was observed especially in the latter two sessions.

The change of ERD/ERS values across the three sessions were analyzed and are illustrated in Figure 6A (calculated from all the trials) and Figure 7A (calculated from only the hit trials), respectively. The first row shows the ERD/ERS value of channel C3 and C4 during the right-hand motor imagery task and the second row displays the ERD/ERS value of channel C3 and C4 during the left-hand motor imagery task. The ERD/ERS results from all the trials were very similar to the results from only the hit trials. Apparently, from the group level, there was a strong ERS for channel C4 compared to channel C3 during the right-hand motor imagery task after the display of the target cue – the ERS increased in general after the display of the target cue and continued to increase after the display of the cursor. On the other hand, the ERS was stronger for channel C3 compared to channel C4 during the left-hand motor imagery task. The black thick bar and the blue thick bar on the *x*-axis depicted the starting cue of target display and the starting time point of the cursor movement feedback, respectively. The gray shaded rectangular area marked the period when the ERD/ERS value of channel C3 and C4 were selected and averaged to compare the ERD/ERS values among session pairs. These results are plotted in Figure 6B for all trials and Figure 7B for only the hit trials. A mixed repeated measures ANOVA [2 (Channel: C3/C4) × 3 (sessions)] was used to determine whether the ERD/ERS values changed over time for channel C3 and C4 calculating from all the trials in Figure 6B and only the hit trials in Figure 7B. The statistical analysis (Right hand task, main effect channel: *F*_1,82_ = 8.67, *p* = 0.004, η^2^ = 0.07; main effect sessions: *F*_2,164_ = 1.57, *p* = 0.21, η^2^ = 0.005; channel × session interaction: *F*_2,164_ = 1.90, *p* = 0.153, η^2^ = 0.006; Left hand task, main effect channel: *F*_1,82_ = 10.39, *p* = 0.002, η^2^ = 0.097; main effect sessions: *F*_2,164_ = 0.50, *p* = 0.61, η^2^ = 0.0009; channel × session interaction: *F*_2,164_ = 0.293, *p* = 0.746, η^2^ = 0.0005) in Figure 6B showed that there was no significant main effect over time, but there was a significant main effect for channels. The *post hoc* linear mixed effect models (lme) test (ERD/ERS for Right hand task, S1: C4–C3 = 0.45, SEM = 0.295, *Z*_value_ = 1.54, *p* = 0.13; S2: C4–C3 = 0.70, SEM = 0.20, *Z*_value_ = 3.58, *p* = 0.0003; S3: C4–C3 = 0.90, SEM = 0.29, *Z*_value_ = 3.11, *p* = 0.002; ERD/ERS for Left hand task, S1: C4–C3 = -0.72, SEM = 0.292, *Z*_value_ = -2.46, *p* = 0.014; S2: C4–C3 = -0.70, SEM = 0.220, *Z*_value_ = -3.19, *p* = 0.0014; S3: C4–C3 = -0.82, SEM = 0.227, *Z*_value_ = -3.62, *p* = 0.0003) revealed that there was a significant difference of ERD/ERS values between channel C3 and channel C4 for both session 2 and session 3 during the right hand motor imagery task. There was a significant difference for all the three sessions during the left-hand motor imagery task. Similarly, the statistical analysis results (Right hand task, main effect channel: *F*_1,82_ = 17.97, *p* = 0.00006, η^2^ = 0.14; main effect sessions: *F*_2,164_ = 2.12, *p* = 0.12, η^2^ = 0.007; channel × session interaction: *F*_2,164_ = 0.89, *p* = 0.413, η^2^ = 0.003; Left hand task, main effect channel: *F*_1,82_ = 22.39, *p* = 0.000009, η^2^ = 0.17; main effect sessions: *F*_2,164_ = 0.084, *p* = 0.92, η^2^ = 0.0002; channel × session interaction: *F*_2,164_ = 0.342, *p* = 0.71, η^2^ = 0.0009) in Figure 7B showed that there was no significant main effect over time, but there was a significant main effect for channels. The *post hoc* linear mixed effect models (lme) test (ERD/ERS for Right hand task, S1: C4–C3 = 0.91, SEM = 0.300, *Z*_value_ = 3.03, *p* = 0.0024; S2: C4–C3 = 1.04, SEM = 0.23, *Z*_value_ = 4.61, *p* = 0.000004; S3: C4–C3 = 1.25, SEM = 0.33, *Z*_value_ = 3.78, *p* = 0.0002; ERD/ERS for Left hand task, S1: C4–C3 = -1.07, SEM = 0.296, *Z*_value_ = -3.60, *p* = 0.0003; S2: C4–C3 = -0.91, SEM = 0.197, *Z*_value_ = -4.60, *p* = 0.000004; S3: C4–C3 = -0.98, SEM = 0.194, *Z*_value_ = -5.08, *p* = 0.0000004) revealed that there was a significant difference of ERD/ERS values between channel C3 and channel C4 for each session and motor imagery task for each hand. Besides the ERD/ERS values of channel C3 and channel C4, the group average ERD% lateralization calculated from all the trials with respect to the hand task, across the three training sessions are shown in Figure 8A. From the visual inspection, there was an increasing trend of lateralization for the right-hand task but an opposite decreasing trend of lateralization for the left-hand task. The statistical analysis for the grand average ERD lateralization values during the period of [5–7.5] s is shown in Figure 8B. A 2 (imagination task: right hand vs. left hand) × 3 (sessions) ANOVA was performed to evaluate the effect of training and imagination type. We observed a significant main effect of imagination type (*F*_1,82_ = 7.85, *p* = 0.006, η^2^ = 0.06). No significant main effect of sessions (*F*_2,164_ = 0.22, *p* = 0.80, η^2^ = 0.0008) and imagination type × sessions interaction (*F*_2,164_ = 3.13, *p* = 0.047, η^2^ = 0.01, Greenhouse-Geisser correction for violations of sphericity, *p* = 0.054). The *post hoc* linear mixed effect models (lme) were performed between imagination types in each session. In sessions 2 and 3, significant ERD lateralization was found between the right-hand task and left-hand task (S2: *Z*_value_ = 2.84, *p* = 0.004; S3: *Z*_value_ = 3.15, *p* = 0.002). Additionally, the log power spectra difference between channel C3 and channel C4 of five frequency bands (1–7, 7–10, 10–14, 14–28, and 29–40 Hz) across three training sessions are illustrated in Figure 9. A 5 (frequency bands) × 3 (sessions) ANOVA was performed to evaluate the effect of training and frequency band. The statistical analysis showed a significant main effect of frequency band (*F*_4,205_ = 4.52, *p* = 0.002, η^2^ = 0.07), a significant main effect of sessions (*F*_2,410_ = 3.56, *p* = 0.03, η^2^ = 0.002) and no significant imagination type × sessions interaction (*F*_8,410_ = 0.32, *p* = 0.96, η^2^ = 0.0008). Thus, we performed the *post hoc* linear mixed effect models (lme) test for both frequency bands and the training sessions. A significant difference was found in session one between the log power difference in 10–14 and 1–7 Hz (Log power difference, S1: P_10-14 Hz_ – P_1-7 Hz_ = 0.12, SEM = 0.037, *Z*_value_ = 3.29, *p* = 0.009), in session 2 between the log power difference in 10–4 and 1–7 Hz, in 14–28 and 1–7 Hz, in 29–40 and 1–7 Hz (Log power difference, S1: P_10-14 Hz_ – P_1-7 Hz_ = 0.13, SEM = 0.031, *Z*_value_ = 4.02, *p* < 0.001; P_14-28 Hz_ – P_1-7 Hz_ = 0.10, SEM = 0.031, *Z*_value_ = 3.34, *p* = 0.007; P_29-40 Hz_ – P_1-7 Hz_ = 0.11, SEM = 0.031, *Z*_value_ = 3.59, *p* = 0.003), in session 3 between the log power difference in 10–14 and 1–7 Hz, in 14–28 and 1–7 Hz, in 29–40 and 1–7 Hz (Log power difference, S1: P_10-14 Hz_ – P_1-7 Hz_ = 0.13, SEM = 0.034, *Z*_value_ = 3.73, *p* = 0.002; P_14-28 Hz_ – P_1-7 Hz_ = 0.11, SEM = 0.034, *Z*_value_ = 3.10, *p* = 0.017; P_29-40 Hz_ – P_1-7 Hz_ = 0.11, SEM = 0.034, *Z*_value_ = 3.10, *p* = 0.016). No significant difference was found across the three training sessions in any of the five frequency bands after the *post hoc* lme test. Since the group average results of ERD/ERS might mask some individual changes, four individual examples of the time varying average ERD/ERS values across the three training sessions were shown for the right-hand task and left-hand task, separately, in Figure 10 and Supplementary Figures S1–S3. In Figure 10, subject 3 showed a strong ERD on the contralateral hemisphere and a moderate ERS on the ipsilateral hemisphere especially in the latter two sessions for both the right-hand task and the left-hand task. In Supplementary Figure S1, subject 18 showed a moderate ERD on the contralateral hemisphere but a very strong ERS on the ipsilateral hemisphere. In Supplementary Figure S2, subject 34 displayed strong but separable ERSs on both contralateral and the ipsilateral hemisphere. In Supplementary Figure S3, a very weak ERD on the contralateral hemisphere and a very strong ERS on the ipsilateral hemisphere was observed in all three sessions for subject 25.

## Discussion

From the results of BCI behavioral performance, we were able to see a significant increase of group average PVC in the third session (78.3%) compared to the first session (72.0%), but the increase was not significant on the second session. Zich et al. (2015a) conducted an experiment consisting of four sessions on four consecutive days. They reported an improvement of online classification accuracy in a group of subjects (*N* = 16) from day 2 (69.1%) to day 4 (73.3%) but did not find any significant change from day 2 to day 3 either. Our results were in accordance with theirs in general, except that their subjects were exposed to an additional training session with a high-density EEG system on the first day. Due to the change of electrodes used for each individual from day 1 to the remaining sessions, in their results (Zich et al., 2015a), the classification accuracy of day 1 was not compared to the remaining three sessions. By contrast, our results clearly showed that training induced significant improvement of classification accuracy from the first session to the third session in a larger population size (*N* = 42). Additionally, a marginally significant improvement of ITR from the first session to the third session was also shown in this study. Note that the significance was only reached in the low performance group when dividing the population into the high and low BCI performers according to their PVC accuracy, even though the high BCI performers showed a similar trend of increasing group average performance. Kaiser et al. (2014) found that a significant increase of [oxy-Hb] in fNIRS and a stronger ERD in the upper beta-frequency (24–30) band of the EEG were only shown in the low BCI performers (five subjects) after ten sessions of BCI training. Perdikis et al. (2018) showed an extensive training course of more than ten BCI sessions in two spinal cord injuries (SCI) pilots. They found a gradual improvement of both behavioral performance and SMR modulation when contrasting the results of the first training session and last training session under real-world and even adverse conditions. In our study, a significant improvement of behavioral performance (PVC and ITR) was only visible in the low BCI performance group (18 subjects) as well, but no significant neurophysiological change, such as *R*^2^ values and ERD/ERS, was observed. This behavioral improvement is due to the larger population size, but the insignificant neurophysiological change is perhaps a result of fewer training sessions in this study. Nevertheless, the present results indicate that an adequate amount of training is necessary for the success of BCI control, especially for some subjects. For example, in 8 out of 42 subjects (around 20% of subjects), their PVC was below 70% on the first session but improved above 70% in the third session. They would be identified as BCI illiterate (Guger et al., 2003; Blankertz et al., 2010) simply due to an insufficient amount of BCI training and this improvement could happen in just 3–4 h’ training across a few days.

The abort rate reflected how easily subjects could finish the task confidently and effectively. In the abort trials where subjects could neither hit the correct target nor the incorrect one, the subjects could not effectively modulate their brain rhythms to reach the target. For some subjects, the decrease of abort rate could be dramatic. For example, subject 19 had an abort rate of 14.2% in the first session but decreased the abort rate to 3.3% by the third session; subject 33 had an abort rate of 88% in the first session but decreased the abort rate to 56%. However, the group average did not show any significant difference. The abort rate was not decreased significantly from session 1 to session 3 because most subjects did not show a consistent change of abort rates across the sessions when individual results were investigated. This might be due to the abort rate not being an explicit measure/goal which subjects intentionally had to reduce. It might be worth investigating the potential effect on the change of these measures by displaying the PVC and abort rates to the subjects at the end of each run. For high BCI performers, there were only 6/24 subjects whose abort rates were higher than 50% in the third session, but all were below 60% with a single exception of 68%. For the poor performers, there were only 2/18 subjects whose abort rates were lower than 50%; all the other poor performers had abort rates within the range of (50–80%) in the third session. Thus, the high BCI performers could generally complete the task relatively well and confidently. However, the low BCI performers struggled to complete the task. This separation of high BCI performers and poor BCI performers is in accordance with previous BCI research literature.

Similarly, the feedback duration could effectively show another important metric measuring subjects’ modulation of brain rhythm to finish the tasks to a certain degree. The shorter the feedback duration toward the ideal 3 s, the better the subject’s modulation is. Although there are a few cases where the subjects’ average feedback duration reduced more than 1 s in session 3 compared to their session 1, the group average did not show any significant difference. Not surprising, most subjects did not show a consistent change of feedback duration across the sessions when individual results were checked. We hypothesize that this might be because the feedback duration was also not an explicit goal for subjects to intentionally reduce. There was no explicit reward on this measure. Thus, a significant change in feedback duration could not happen in the current training setup (a few hours). Because we did not find a consistent change of neurophysiological signatures across sessions, another possibility for this insignificant change was insufficient training sessions for most subjects. A more delicate experiment needs to be conducted to address this question of the insignificance of metrics (abort rate, feedback duration) for BCI learning. The analysis of Riemannian class distance provides distinctiveness information. This metric measures the difference of EEG patterns by calculating the Riemannian distance of class covariance from all the channels between the two imagination tasks. No statistically significant difference was observed from any session pair although a slightly increasing trend was visible. This metric might not be sensitive enough to capture the change if there was any. This insensitivity was perhaps because all the channels were included in this analysis. The ITR is another frequently used metric to measure the efficiency of an online BCI system. In the current study, we found a marginal improvement of ITR in a group of 42 subjects. Again, only the low BCI performers approached a significant improvement of ITR when the subjects were divided into high and low BCI performance groups as previously done for the PVC analysis. This ITR result was in accordance with the PVC result, and it further showed that not only did subjects’ performance accuracy improve during the training, but their efficiency did as well. Grouping subjects into high and low BCI performers further revealed that the training was particularly crucial to the low BCI performers.

From the results of the *R*^2^ values for channel C3 and C4, we did not observe a significant change of *R*^2^ value for channel C3 across the three training sessions. No clear trend was shown for channel C4 either. This observation held, regardless of whether all trials, or only the hit trials were used. However, the statistics did show a significant difference of the *R*^2^ value between channel C3 and channel C4 at the beginning of the training session, i.e., session 1, if only the hit trials were used. This difference between the left hemisphere and right hemisphere disappeared at the second and third training session. This might allow us to hypothesize that the contribution of BCI control from channel C3 could become stronger compared to channel C4 across the training progress. However, we did not find any significant change of *R*^2^ values across the sessions for both channel C3 and channel C4. Thus, the hypothesis was not supported by the current data. Since five out of 42 subjects were left-handed, we display the individual *R*^2^ values for both the right-handed subjects and the left-handed subjects separately across the three training sessions in Figure 5. A previous study suggested that handedness (dominant hand vs. non-dominant hand) might reveal the imbalanced ability to modulate brain rhythms of the left hemisphere and right hemisphere (Bai et al., 2005). We did find that most of the right-handed subjects showed a higher *R*^2^ value on the right hemisphere of channel C4 while the opposite phenomenon was observed for most left-handed subjects across the three training sessions. This observation was masked by the group analysis of merging the right-handed and the left-handed subjects. However, whether the right-handed subjects have a dominant modulation, i.e., larger *R*^2^ values, on the right hemisphere of channel C4, or the left-handed subjects have a dominant left side modulation over channel C3, did not pass the significance test. It is worth noting that there were only five left-handed subjects in this study; it is hard to make any conclusion on the left-handed subjects.

From the result of the ERD/ERS value for left- and right-hand tasks, we could see clear and strong ipsilateral ERS compared to the EEG activity of the baseline. In this study, most of the subjects who showed the ability of effective BCI control demonstrated the strong modulation of ERS on the ipsilateral hemisphere with respect to the imagination task. The group average results supports this observation. Several individual examples such as subject 18 in Supplementary Figure S1, subject 34 in Supplementary Figure S2, and subject 25 in Supplementary Figure S3 showed strong ERS modulation. In previous studies (Pfurtscheller et al., 2006; Zich et al., 2015a), Pfurtscheller et al. showed strong bilateral ERDs in their group analysis which were different from ours. This might be due to the difference of the cursor control applications and decoding algorithms since our previous study showed a consistent ERS modulation as well (Meng et al., 2016). The average ERD/ERS values during the period of the first 2.5 s after the cursor’s movement showed a significant difference in the electrode C3 and C4 for both left-hand and right-hand tasks. This indicated that the strong ipsilateral ERS drove the cursor in the same direction as the imagination of the corresponding hand. But the statistical analysis showed that there was no significant change of ERD/ERS values across the three training sessions for either channel C3 or channel C4, for either the right-hand or left-hand tasks. Bai et al. (2005) previously concluded that the activation on the left hemisphere during left-hand movements is greater than that on the right hemisphere during right-hand movements, in a group of nine right-handed subjects; they also reported that a contralateral preponderant ERD distribution for right-hand movements, i.e., lateralized power was only observed during preparation of right-hand movement, while no lateralized power was seen during the preparation of left-hand movement. Our ERD lateralization analysis in Figure 8 is in line with their findings although our task was motor imagination. An obvious ERD lateralization and a trend of increasing ERD lateralization was only found in the right-hand imagination task; a negligible ERD lateralization was displayed in the left-hand imagination task. Moreover, a significant difference of ERD lateralization was found between the right- and left-hand imagination task during both sessions 2 and 3. Zich et al. (2015a) showed a dominant role of alpha (8–12 Hz) modulation and a significant increase of log power difference in both the alpha and beta (13–30 Hz) bands in their study. We did a similar group analysis in Figure 9. A similar dominant role of alpha power (10–14 Hz in our case) was found, but we did not find any significant improvement of a log power difference in our data.

### Limitations and Future Works

The combined analysis of two studies gives us a pool of 42 subjects and a unique opportunity to study the learning effect across training sessions. However, there were only three training sessions for each subject. We did see some significant change of behavioral performance such as the improvement of PVC and ITR, and other trends of neurophysiological results including the increase of ERD lateralization for right-hand task although they were not statistically significant yet. It is not clear yet when the change of behavioral performance might saturate and when significant changes of neurophysiological characteristics such as the *R*^2^ value, ERD lateralization might emerge and saturate in this study. Also, the session interval for each participant varied between 1 day to 1 week due to scheduling conflicts for a large number of subjects. This might introduce an extra variance to the result. In the future, it is worth conducting a study that consists of more BCI sessions in a large number of subjects with more controllable session intervals. Furthermore, other metrics like Kullback-Leibler (KL) divergence might help to explore the change of data distribution within and across the sessions (Vidaurre et al., 2011) and it might extract some additional information which might help optimize the experiment in the future.

## Conclusion

In this study, we analyzed a pooled dataset consisting of 42 subjects’ three BCI training sessions. The behavioral performance results showed that there was a significant increase of BCI PVC accuracy (*p* = 0.004) and a marginal significant improvement of ITR (*p* = 0.05) in the third training session compared to the first session. No other significant difference of behavioral measures such as group average abort rate or feedback duration was found across the training sessions. Further analysis on the group average *R*^2^ value indicated that there was a significant difference of the *R*^2^ value on the first training session, but this difference diminished for the remaining sessions if only hit trials were considered; there was no significant difference if all of the trials were used. A significant difference of ERD/ERS values between the channel C3 and C4 was shown across the three training sessions for both left-hand and right-hand tasks, and this stronger ipsilateral ERS phenomenon explains the experimental observation of the successful control of the cursor toward the same side of hand imagination. A significant ERD lateralization was only shown in the right-hand imagination task but not in the left-hand imagination task in the group level. Neither *R*^2^ values nor ERD/ERS values showed significant change across the three training sessions for neither channel C3 nor channel C4. Altogether, the results of this study revealed the importance of training for successful BCI control and that a significant improvement in BCI skills could happen with just a few hours of training, distributed over a few days. Therefore, experimental design leveraging an engaging training protocol might be critical in making a successful BCI application in the future.

## Data Availability

The de-identified datasets generated for this study can be found online at http://dx.doi.org/10.6084/m9.figshare.7959572.

## Ethics Statement

All procedures and protocols were approved by the Institutional Review Boards of the University of Minnesota and Carnegie Mellon University. Written informed consents were obtained from all of the subjects before their participation in the experiment.

## Author Contributions

JM wrote the first draft of the manuscript, performed the research, and analyzed the data. JM and BH edited the manuscript, conceived and designed the experimental paradigm, and wrote the manuscript.

## Conflict of Interest Statement

The authors declare that the research was conducted in the absence of any commercial or financial relationships that could be construed as a potential conflict of interest.

## Supplementary Material

Figure S1 An individual example (Subject 18) of the time varying average ERD/ERS value for the right hand task (the first row) and the left hand task (the second row) across the training sessions. Shown is the similar convention to the group average results. A moderate ERD on the contralateral hemisphere and a very strong ERS on the ipsilateral hemisphere was observed in all of three sessions.

Figure S2 An individual example (Subject 34) of the time varying average ERD/ERS value for the right hand task (the first row) and the left hand task (the second row) across the training sessions. Shown is the similar convention to the group average results. Strong but separable ERSs on both of the contralateral and the ipsilateral hemisphere were observed especially in the latter two sessions.

Figure S3 An individual example (Subject 25) of the time varying average ERD/ERS value for the right hand task (the first row) and the left hand task (the second row) across the training sessions. Shown is the similar convention to the group average results. A very weak ERD on the contralateral hemisphere and a very strong ERS on the ipsilateral hemisphere was observed in all of three sessions.
