# Supplementary figures and images for: Exploring Training Effect in 42 Human Subjects Using a Non-invasive Sensorimotor Rhythm Based Online BCI
Source: Front Hum Neurosci. 2019 Apr 17;13:128. doi: 10.3389/fnhum.2019.00128 (PMC6481252; doi:10.3389/fnhum.2019.00128)

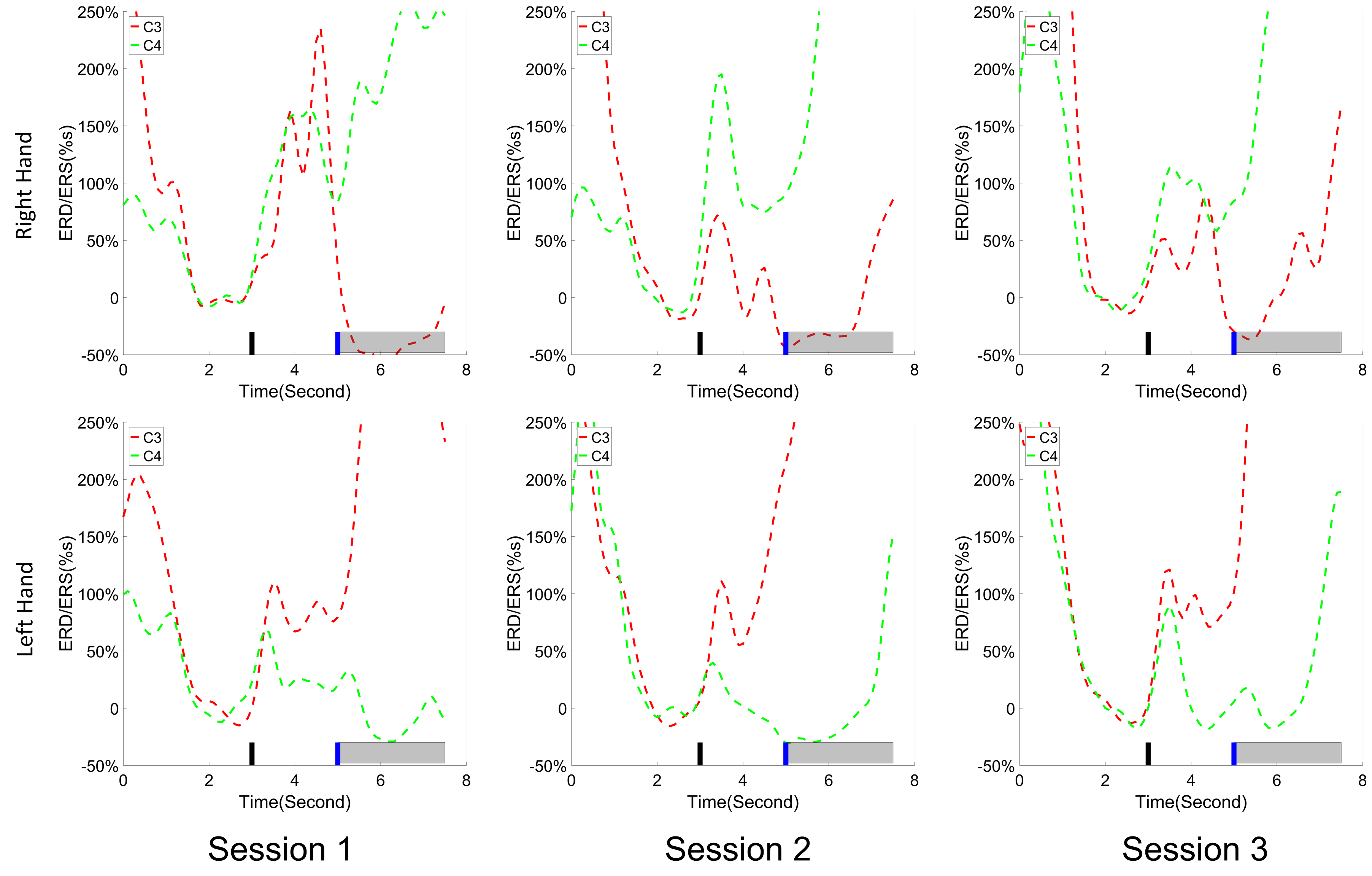

Supplement: Figure S1 — An individual example (Subject 18) of the time varying average ERD/ERS value for the right hand task (the first row) and the left hand task (the second row) across the training sessions. Shown is the similar convention to the group average results. A moderate ERD on the contralateral hemisphere and a very strong ERS on the ipsilateral hemisphere was observed in all of three sessions. [file Image_1.TIFF]

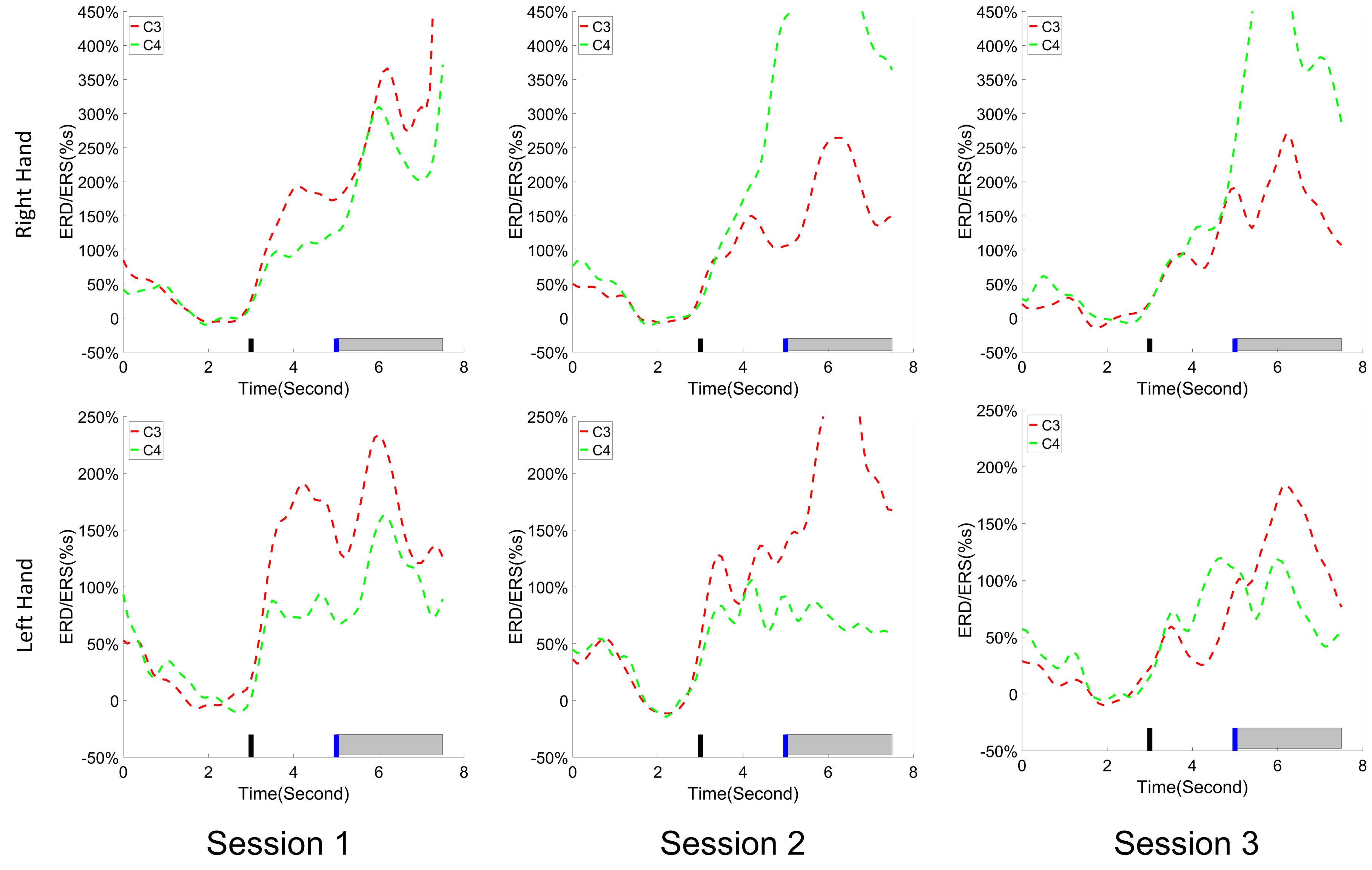

Supplement: Figure S2 — An individual example (Subject 34) of the time varying average ERD/ERS value for the right hand task (the first row) and the left hand task (the second row) across the training sessions. Shown is the similar convention to the group average results. Strong but separable ERSs on both of the contralateral and the ipsilateral hemisphere were observed especially in the latter two sessions. [file Image_2.TIFF]

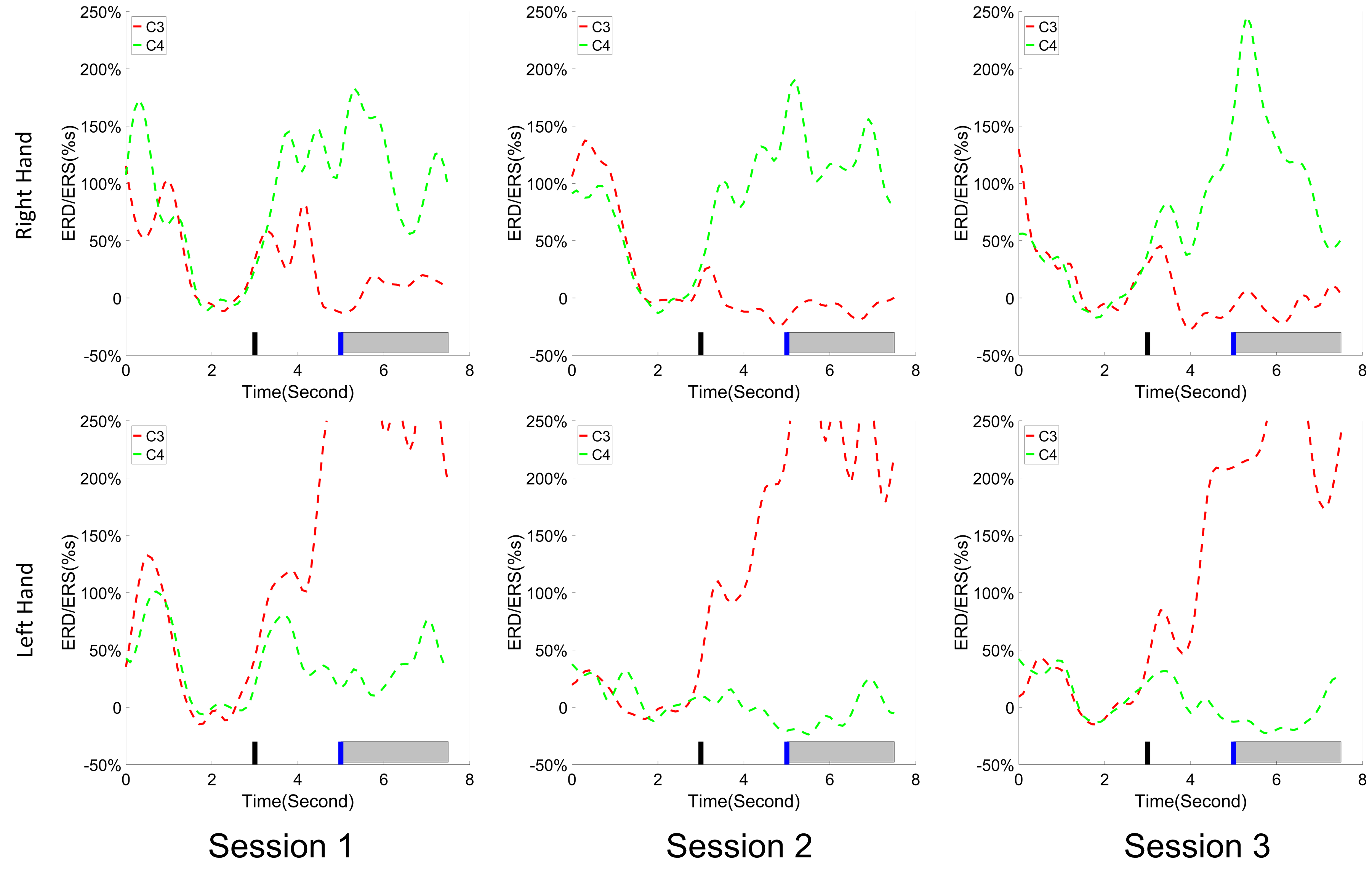

Supplement: Figure S3 — An individual example (Subject 25) of the time varying average ERD/ERS value for the right hand task (the first row) and the left hand task (the second row) across the training sessions. Shown is the similar convention to the group average results. A very weak ERD on the contralateral hemisphere and a very strong ERS on the ipsilateral hemisphere was observed in all of three sessions. [file Image_3.TIFF]
